# A tutorial for conducting intersectional multilevel analysis of individual heterogeneity and discriminatory accuracy (MAIHDA)

**DOI:** 10.1016/j.ssmph.2024.101664

**Published:** 2024-03-26

**Authors:** Clare R. Evans, George Leckie, S.V. Subramanian, Andrew Bell, Juan Merlo

**Affiliations:** a Department of Sociology, University of Oregon, Eugene, OR, USA; b Centre for Multilevel Modelling and School of Education, University of Bristol, UK; c Department of Social and Behavioral Sciences, Harvard T.H. Chan School of Public Health, Boston, MA, USA; d Harvard Center for Population and Development Studies, Cambridge, MA, USA; e Sheffield Methods Institute, University of Sheffield, Sheffield, UK; f Research Unit of Social Epidemiology, Faculty of Medicine, University of Lund, Sweden; g Center for Primary Health Care Research, Region Skåne, Malmö, Sweden

**Keywords:** MAIHDA, Intersectionality, Multilevel models, Health inequality, Linear regression, Logistic regression, Quantitative methods, Social determinants

## Abstract

Intersectional multilevel analysis of individual heterogeneity and discriminatory accuracy (I-MAIHDA) is an innovative approach for investigating inequalities, including intersectional inequalities in health, disease, psychosocial, socioeconomic, and other outcomes. I-MAIHDA and related MAIHDA approaches have conceptual and methodological advantages over conventional single-level regression analysis. By enabling the study of inequalities produced by numerous interlocking systems of marginalization and oppression, and by addressing many of the limitations of studying interactions in conventional analyses, intersectional MAIHDA provides a valuable analytical tool in social epidemiology, health psychology, precision medicine and public health, environmental justice, and beyond. The approach allows for estimation of average differences between intersectional strata (stratum inequalities), in-depth exploration of interaction effects, as well as decomposition of the total individual variation (heterogeneity) in individual outcomes within and between strata.

Specific advice for conducting and interpreting MAIHDA models has been scattered across a burgeoning literature. We consolidate this knowledge into an accessible conceptual and applied tutorial for studying both continuous and binary individual outcomes. We emphasize I-MAIHDA in our illustration, however this tutorial is also informative for understanding related approaches, such as *multicategorical MAIHDA*, which has been proposed for use in clinical research and beyond. The tutorial will support readers who wish to perform their own analyses and those interested in expanding their understanding of the approach. To demonstrate the methodology, we provide step-by-step analytical advice and present an illustrative health application using simulated data. We provide the data and syntax to replicate all our analyses.

## Highlights

• MAIHDA is a new gold standard for quantitative intersectional analysis of inequity.
• This is a tutorial on MAIHDA analysis with downloadable code and simulated data.
• Step-by-step guidance is provided for fitting linear and logistic MAIHDA models.
• We consolidate conceptual, technical and practical advice from the literature.
• Downloadable code with examples in alternative software is also available online.

## 1 Introduction

Intersectional multilevel analysis of individual heterogeneity and discriminatory accuracy, also called intersectional MAIHDA or I-MAIHDA, is an innovative approach for investigating inequalities in health, disease, psychosocial, socioeconomic, and other outcomes (Evans, 2015; Evans, Williams, Onnela, & Subramanian, 2018). I-MAIHDA is an application of the broader MAIHDA approach for quantifying inequalities within an intersectional framework. While interest in quantitative intersectionality has been growing in population health and other fields, widespread adoption has been hampered by limitations of conventional modeling approaches. I-MAIHDA and related approaches, such as multicategorical MAIHDA (Evans, 2024; Jones, Johnston, & Manley, 2016) and geographical MAIHDA (Merlo, 2014; Merlo, Wagner, and Leckie 2019), address many of these limitations, and as such they have the potential to make quantitative intersectional and multicategorical analyses more accessible, including in health and education inequalities research, precision medicine and public health, environmental justice scholarship, and across the social sciences.

MAIHDA has conceptual, methodological, and theory-oriented advantages over conventional single-level regression analysis (Bell, Holman, & Jones, 2019; Evans, 2019a; Evans et al., 2018; Evans, Leckie, and Merlo 2020) and over other quantitative approaches for investigating intersectional effects (Mahendran, Lizotte, & Bauer, 2022a; 2022b). Intersectional MAIHDA provides an ideal analytical instrument in precision public health (Olstad & McIntyre, 2019; Persmark, Wemrell, Zettermark, et al., 2019), and answers calls for innovative approaches that consider both differences between group averages and variation within these groups around their average values (Merlo, 2014; Merlo & Wagner, 2013). Due to its practical-methodological and theory-oriented advantages over conventional methods, I-MAIHDA has been hailed as “the new gold standard for investigating health disparities in (social) epidemiology” (Merlo, 2018), clinical and biomedical research (Evans, 2024), and beyond.

The term “MAIHDA” predates the specific statistical approach (I-MAIHDA) it is now widely associated with (and which is the focus of this tutorial) (Merlo, 2014). Originally, MAIHDA referred to a reorganization of existing multilevel modelling concepts, in order to systematically consider both differences between group averages and individual heterogeneity around those averages. While historically, “MAIHDA” involved fitting multilevel models of individuals nested hierarchically in schools, neighbourhoods, workplaces, or similar concrete contexts, with particular emphasis on geographic inequalities in health (Merlo, 2014; Merlo, Wagner, & Leckie, 2019), the new statistical approach associated with the term broadens this conceptualization of ‘context.’

First proposed in 2015 by Evans (Evans, 2015) (later published as: Evans et al. (2018)), and soon after explored by Jones et al. (2016), and Bell et al. (2019), MAIHDA now refers to the use of multilevel models to examine patterns of inequalities across social strata, constructed through high-dimensional, multicategorical cross-tabulation of identities, conditions, or other factors. When applied in an intersectional framework, the approach is more specifically called I-MAIHDA, and it is used to examine inequalities by factors such as gender, race/ethnicity, and socioeconomic status. A more general application, multicategorical MAIHDA, has also been proposed, with applications in fields such as clinical/biomedical research (Evans, 2024; Rodriguez-Lopez, Leckie, Kaufman, & Merlo, 2023). Specifically, Evans (2024) has argued that MAIHDA should guide study design across the clinical and health sciences for examining high-dimensional effect measure modification, interactions, and subgroup comparisons. Though we focus here on I-MAIHDA, this tutorial can be used to inform and guide more general MAIDHA research, including multicategorical MAIHDA.

Following Evans et al. (2018), we prefer the term “social strata” when referring to analytic subgroups, such as at particular intersections of gender/race/class, because it evokes the *provisional* adoption of labels for stratified analyses, while remaining skeptical of the term ‘group’ (Brubaker, 2002) and avoiding conceptualizing labels as monolithic, unchanging, or inflexible. In MAIHDA, stratum inequalities are not only evaluated in terms of differences between stratum averages, but by informing on the share of the total heterogeneity in the outcome that is at the stratum level. In this way MAIHDA enhances the focus on population health rather than on individual biomedical susceptibilities, as may be the case in precision medicine. It also avoids the well-known “tyranny of the averages” problem (Merlo, 2014; Merlo & Wagner, 2013), where group averages are over-emphasized and consideration of variance or heterogeneity is under-attended to.

The new I-MAIHDA approach has already been applied to examine a variety of health inequalities, including body mass index (Evans et al., 2018; Hernández-Yumar et al., 2018), COPD risk (Axelsson Fisk et al., 2018), depression (Evans & Erickson, 2019), prescription opioid (mis)use (Persmark, Wemrell, Evans, et al., 2019; Persmark, Wemrell, Zettermark, et al., 2019), biomarkers of healthy aging (Holman, Salway, & Bell, 2020), use of hormonal contraception and risk of depression (Zettermark et al., 2021), and HPV vaccination (Zubizarreta et al., 2022). To date, MAIHDA (and especially I-MAIHDA) have primarily been developed and used to study health inequalities. However, depending on the specific outcomes and strata definitions chosen by researchers, MAIHDA has broad uses and appeal across the social sciences, as well as in medicine and related fields. MAIHDA’s recent use in related social science disciplines, such as sociology of sexualities (Silva & Evans, 2020), environmental justice (Alvarez, Calasanti, Evans, & Ard, 2022; Alvarez & Evans, 2021), and education (Keller, Oliver, Preckel, & Brunner, 2023; Prior, Evans, Merlo, & Leckie, 2022; Prior and Leckie 2023), hint at its larger, cross-disciplinary potential.

Specific advice for conducting and interpreting intersectional and multicategorical MAIHDA models has been scattered across a burgeoning literature (Axelsson Fisk et al., 2018; Bell et al., 2019; Evans, 2015, 2019c, 2019a, 2024; Evans et al., 2018, 2023, 2020; Green, Evans, & Subramanian, 2017; Keller et al., 2023; Merlo, 2018; Wemrell, Mulinari, & Merlo, 2017). Consequently, researchers interested in applying this approach may be seeking a resource with detailed practical advice as well as general conceptual information. Addressing this need, we provide a conceptual and applied tutorial for conducting intersectional analyses for both continuous and binary individual outcomes using MAIHDA.

We provide detailed annotated code for analysis in both Stata and R, using maximum likelihood estimation (MLE), and a simulated practice data set. Additional code resources, such as syntax for estimation using other approaches and software (e.g., Markov Chain Monte Carlo (MCMC) estimation in Stata and MLwiN) will be made available in an online repository: https://doi.org/10.17605/OSF.IO/DTVC3. All intersectional MAIHDA models in this tutorial are two-level random-intercept models, and readers will be able to fit them using most statistical analysis software. Interested users can follow along with this tutorial and learn how to apply MAIHDA in their own research. This tutorial is also intended for readers who wish to expand their general understanding of the intersectionality framework and MAIHDA methodology.

### 1.1 The meaning of “interaction” in intersectionality versus regression analysis

From the outset it is important to distinguish between “interaction” within intersectionality as a theoretical framework and “interaction” in regression analysis. Intersectionality is a theoretical framework which emerged from critical, Black feminist scholarship (Cho, Crenshaw, and McCall 2013; Collins, 1990, 2009; Crenshaw, 1989). As a critical theory, intersectionality draws attention to the ways in which systems of oppression such as sexism, racism, and socioeconomic inequality are interlocking, inseparable, and mutually constituted. Furthermore, it demands reform and transformation of these systems.

Theorized in quantitative terms, individuals can be understood to occupy different positions in intersecting social hierarchies based on their social identities and socioeconomic conditions/class. Though often treated (and measured) as individual-level variables, these identities only take on full meaning in combination and social context, and so we consider social strata and identities as being contextual in nature. Some intersectional contextual identity-positions will experience advantages or disadvantages that are entirely *unique* to their particular intersectional location (e.g., a type of discrimination targeted specifically at Black women). In other cases, occupying a particular intersectional position will increase or decrease the chances of advantageous (or disadvantageous) exposures, opportunities, or outcomes.

Both of these situations differ in important ways from the concept of *interaction effects* in regression analysis. In regression models, an interaction effect captures the extent to which two (or more) unique social exposures combine to create patterns of inequality in an outcome that would not be adequately described using additive effects alone (i.e., main effects). Some scholars distinguish between “interaction” (the joint casual contributions of multiple factors) and “effect measure modification” (where the association between possible cause X and outcome Y will differ across levels of another variable M) (Vanderweele, 2009). For simplicity, we henceforth use the term “interaction” to refer to both.

Translating theory into methods, jointly considering the “double jeopardy” of two marginalized positionalities (Black and woman, for instance) would imply use of additive main effects to describe inequalities. However, this additive treatment would not capture what King called the multiplicative “multiple jeopardy” of being both Black and a woman (King, 1988). For instance, assessing risk of a health outcome additively based on race and gender (e.g., “Black” plus “woman” additive main effects) may not be enough to describe the prevalence of a particular health outcome among those who are Black *and* women, necessitating an additional multiplicative *interaction* term (Black × woman).

Put simply, intersectionality is a framework that enables us to theorize about, and critique, social experiences (*exposures*) at particular intersections and the systems of oppression that create those experiences, while regression analyses that include interactions capture the *effects* of these experiences on particular outcomes. Thus, the effect of exposures may or may not result in statistically significant interaction effects. Failure to find statistically significant interaction effects says nothing about the validity of intersectionality—it neither proves nor disproves the existence of intersectional experiences. In this sense, intersectionality may be better thought of as a *framework* for analysis (e.g., how we structure our questions), rather than a *testable theory*.

### 1.2 Theory in intersectional analysis

Our purpose is to provide a tutorial for researchers interested in understanding and applying intersectional MAIHDA. We will therefore focus on practical methodological issues. However, it is also imperative to engage with theory when using the approach. MAIHDA is a theoretically-oriented and theoretically-driven *descriptive* approach. While future researchers may investigate extensions for MAIHDA, at present it has not been developed or applied for “analytic intersectionality” research, investigating mediation in the production of health inequalities (Bauer & Scheim, 2019). As a related aside, we will henceforth use both the terms *inequality* (differences in outcomes between populations) and *inequity* (differences that are unnecessary or avoidable) throughout this tutorial; We typically theorize differences as inequities, but sometimes refer to inequalities when making more general statements of difference, or to mirror published findings in the literature.

What does it mean for MAIHDA to be a theoretically-oriented and driven descriptive approach? Stated most generally, it is essential to remain clear about what is theorized to drive observed inequities. For instance, in social epidemiology there are numerous theories of population health inequality that speak to the multiplicative nature of disadvantages originating in social determinants, such as fundamental cause theory (Link and Phelan 1995) and ecosocial theory (Krieger, 2011). Researchers should employ theories similar to these, as appropriate to their disciplines, in conjunction with intersectionality to aid in theorizing and interpreting results from MAIHDA. A researcher who provides estimates of inequalities between strata but leaves open the interpretation of what caused them may enable (or invite) a misunderstanding of inequalities as originating in genetic (essentialist) views of race/gender and could unintentionally embolden biological determinist arguments. This is anathema to intersectional and social epidemiologic scholarship in general, both of which have long battled such claims.

While future adaptations and applications of the general MAIHDA approach in other, very different fields with different substantive questions may not require such a focus (e.g., if strata were defined not by race/class/gender but by wholly different variables (Evans, 2024; Rodriguez-Lopez et al., 2023)), we caution scholars working in social epidemiology and related disciplines to strive to situate their interpretations and (often implicit) causal theoretical frameworks *explicitly* within theories of social determinants and an understanding of power structures. Stated differently, health inequalities are produced by complex, multifaceted, overlapping and interacting social processes of embodiment, including material deprivation, psychosocial pathways, and adverse exposures (Krieger, 2011). Explicit attribution of health inequities to unequal power structures and systems of oppression will also help to overcome another issue with some descriptive intersectional scholarship—namely, that atheoretical exercises risk flattening intersectionality from a rich theoretical framework to an analytic approach which is “merely” about the estimation of interaction effects (Evans, 2019b; May, 2015).

## 2 Illustrative example

### 2.1 Simulated data

We demonstrate how to conduct an intersectional MAIHDA (I-MAIHDA) analysis of simulated continuous HbA1c data (linear regression MAIHDAs) and binary diabetic status (logistic regression MAIHDA) using maximum likelihood estimation (MLE) in Stata and R. In order to construct a realistic example for this tutorial, we created a fictitious, simulated practice data set (available in the online Supplementary materials). The simulated data structure is based loosely on Wave 2 of the National Epidemiologic Survey on Alcohol and Related Conditions (NESARC) (Grant and Kaplan 2005), to attain an illustrative example with realistic-looking sample sizes, sociodemographic survey items, and sample distribution across intersectional strata (see Table 1). The original NESARC was a longitudinal study begun in 2001 by the U.S. National Institute on Alcohol Abuse and Alcoholism, and it was designed to survey a representative sample of the U.S. non-incarcerated civilian population, including citizens and non-citizens, aged 18 years and older who were currently residing in the U.S. Wave 2 data was collected between 2004 and 2005. Following the construction of strata used by Evans et al. (2018), we defined 384 possible intersectional strata (=2 sex/gender categories × 3 race/ethnicity categories × 4 education categories × 4 income categories × 4 age categories) with a total of N = 33,000 individuals. For realism, we simulated some strata to be very small (e.g., N < 10) while others are substantially larger.

**Table 1:** Descriptive statistics of individual observations (N = 33,000) and intersectional strata (n = 384) in the simulated data.

Sample Statistics	N		%			
Total	33,000		100			
Sex						
Male	13,931		42.2			
Female	19,069		57.8			
Race/Ethnicity						
White Non-Hispanic	20,075		60.8			
Black Non-Hispanic	6572		19.9			
Hispanic/Latino	6353		19.3			
Education						
Less than high school	5272		16.0			
Completed high school	9097		27.6			
Some college no degree	7080		21.5			
College degree or more	11,551		35.0			
Income (% Poverty Threshold)						
Low income (<100%)	7712		23.4			
Low-middle income (100%–199%)	9198		27.9			
High-middle income (200%–399%)	9605		29.1			
High income (400% or more)	6485		19.7			
Age						
18–29 years	4657		14.1			
30–44 years	10,048		30.5			
45–59 years	9213		27.9			
60+ years	9082		27.5			
HbA1c	N	Mean	Median	SD	Min	Max
Individual values	33,000	40.2	39.8	9.9	10.3	101.2
Observed stratum means	384	40.8	40.3	3.9	23.5	53.8
Predicted stratum means	384	40.8	40.5	2.7	34.2	48.1

*Notes*: The mean of the 33,000 individual observations of HbA1c is the “sample mean.” The mean of the 384 observed stratum means is the “grand mean.” The mean of the 384 predicted stratum means from the MAIHDA model is the “precision-weighted grand mean.”

The simulated outcome, HbA1c, is a recreation of the biomarker commonly used in public health and medical research as an indicator of blood glucose control and diabetes (Gillery, 2013). HbA1c is measured continuously in mmol/mol and a value is provided for each simulated individual. Simulated values were generated to produce realistic features of empirical data, such as meaningful between-stratum inequities, large within-stratum-between-individual variance, and realistic distributions of individuals across the strata and the variables use to construct them (Holman et al., 2020). HbA1c was dichotomized to create the variable diabetic (1 = yes, 0 = no), with values ≥48 mmol/mol indicative of a ‘diabetic range’ (Kaiafa et al., 2021). Throughout this tutorial we use both the term ‘diabetic’ (identity-first language) and ‘individuals with diabetes’ (person-first language), due to mixed views within the diabetes community and disability justice community on whether one is preferred over the other.

Additional code resources, such as example syntax in Stata using Bayesian MCMC estimation (with the bayes prefix), and for fitting models in MLwiN from Stata is also provided at: https://doi.org/10.17605/OSF.IO/DTVC3. As updates and code expansions to other programs become available, they will also be posted. While our focus in this tutorial is on *intersectional* MAIHDA, the methods and code illustrated here are equally applicable to the more expansive *multicategorical* MAIHDA.

### 2.2 Assessing variables and constructing strata for intersectional MAIHDA

#### 2.2.1 Assessment of variables

The simulated data file resembles what might exist in an empirical data set. Each row corresponds to one respondent, and each column is a separate variable, with one for each of the five studied social identities/positions. As in the original analysis, ‘sex’ is used in place of ‘gender’ because this was how the data was recorded in the survey. ‘Race’ here refers to self-identified race and ethnicity, but the variable name was simplified; for brevity, we refer to racialized categories as white, Black, and Hispanic. The five variables are coded as follows:

⁃ *Sex*: 1 = Male; 2 = Female.
⁃ *Race*: 1 = white non-Hispanic; 2 = Black non-Hispanic; 3 = Hispanic or Latinx.
⁃ *Education*: 1 = Less than high school; 2 = Completed high school or equivalent; 3 = Some college but no degree; 4 = College degree or more.
⁃ *Income*: 1 = Low income (below 100% of the Federal Poverty Level, FPL, in 2000); 2 = Low-middle income (100%–199% FPL), 3 = High-middle income (200%–399% FPL); 4 = High income (400% or more of the FPL).
⁃ *Age*: 1 = age 18–29 years; 2 = age 30–44 years; 3 = age 45–59 years; 4 = age 60+ years.

For additional details on how these variables were coded, see Evans et al. (2018).

#### 2.2.2 Constructing the strata ID

Selecting which axes of marginalization or inequality to examine in an intersectional analysis (e.g., age, gender, race/ethnicity, income, sexual orientation, disability) should be based on both theoretical and practical considerations. Theoretical considerations include: *What are the major axes of marginalization and/or inequality experienced by this population, and in particular, what may be relevant to the outcome of interest? What has prior research shown to be important?* Practical considerations include: *What axes of marginalization/inequality were actually measured in this data set? What is the quality of those measures? How were responses coded? How large is the sample and how is the sample distributed across possible strata definitions?*

There are many choices to be made when preparing the variables. For instance, income, education, and age may be provided either continuously or with far more categorical levels than presented here. Decisions about what cutoff values to use or which levels to (re)combine should be supported by theory. However, there is a balance between the ideal set up and practical modeling and data limitations. For instance, using eighteen categories for income would create many small strata after intersecting income with race/ethnicity. While MAIHDA can manage small strata better than some other approaches, it does not entirely avoid the small N problem. In this case, including eighteen income levels would be more granular than is desirable (or needed). As discussed by Evans et al. (2018), the categorizations defined above balance issues of substantive interest with practical data limitations to arrive at a reasonable sample size for most strata.

After provisionally selecting the axes and categories to be included, it is important to consider *how well the data available is distributed across all of the possible intersectional strata*. For example, unless a survey intentionally oversamples from some minoritized populations (e.g., transgender or non-binary individuals), there may be too few respondents in these categories and this situation will render many strata small or empty after combining with other axes, such as race/ethnicity or class. While it is always desirous to include minoritized populations, data limitations may restrict the axes or categories that are able to be analyzed. When using large population databases with millions of respondents, as in Persmark et al.’s analysis of the Swedish registries (Persmark, Wemrell, Zettermark, et al., 2019), small strata may be less frequent but the available measures of minoritized identities may be more limited. These factors should be considered in the selection of data to work with and in the final specification of strata definitions.

One useful way to determine whether the sample size is adequate in most intersections is to examine the number of respondents in each stratum using frequency tabulations. While important to consider, these tabulations can also be difficult to assess overall when many strata are evaluated (e.g., 384 strata). In that case, it can be helpful to determine: *What percent of the strata have X or more respondents?* Where *X* = 10, 20, 30, 50 and 100+ respondents (see tutorial code). Table 2 contains these summary statistics for the instructional dataset. In this case, 81% of the strata have twenty or more respondents and 70% have thirty or more, indicating that most strata seem to have sufficient sample size to obtain reliable estimates. Recent simulation studies have investigated linear and logistic MAIHDA under select parameter conditions with sample size as low as N = 10.4 observations on average per stratum and reported robust estimate accuracy (Mahendran et al., 2022a; Mahendran et al., 2022b), suggesting MAIHDA may enable researchers to disaggregate groups more than alternative methods. However, the generalizability of this finding has yet to be thoroughly investigated. If desired, statistical power calculations can be done (using simulations, such as those conducted by (Bell et al., 2019; Mahendran et al., 2022a, Mahendran et al., 2022b) to assist in making choices about strata definitions, though ultimately options may be limited in secondary data (Snijders, 2005).

**Table 2:** Sample Size of Simulated Intersectional Social Strata, defined by respondent sex, race/ethnicity, education, income, and age (n = 384).

Sample Size Per Stratum	Number of Strata	% of Strata
100 or More	107	27.9
50 or More	202	52.6
30 or More	267	69.5
20 or More	311	81.0
10 or More	347	90.4
Less than 10	37	9.6

Also, worth considering is the extent to which some strata may be systematically smaller than others. For instance, do all of the “very small” strata belong to a single minoritized racial/ethnic or gender classification? If this is the case, then the model will still provide estimates for these strata but due to shrinkage of estimates for small strata (which we discuss later), these estimates will tend to be more conservative (i.e., closer to the average predicted by any fixed effects in the model) than estimates for other strata. This may or may not affect the decision to retain this classification in the analysis, but awareness of this issue is important.

Conducting an intersectional MAIHDA analysis requires us to construct a stratum identifier or ID variable. This variable takes a unique value for each stratum. The data are then viewed as having a two-level structure whereby respondents (level 1) are said to be nested within social strata (level 2). Treating intersectional strata as the second level means that we are conceptualizing strata as “contexts,” or literal social positions within a landscape of intersecting hierarchies (as opposed to properties of individuals operating independently), and that we are analyzing them as such in a multilevel framework (Evans, Leckie, & Merlo, 2020).

One way to construct the stratum ID would be to assign values ranging from 1 to J, where J = N, the total number of strata to analyze (N = 384, in this case). However, it may be prudent to attach a meaningful value label to each ID value to facilitate later interpretation and plotting of predicted stratum effects. For example, we might attach the value label “Female, Hispanic, Some college, High income, 30 to 44” for those in that stratum. A disadvantage of this is that the value labels are rather long. Alternatively, we might construct a multi-digit ID code, where each digit position corresponds to one of the axes of marginalization being analyzed. This is the approach we have chosen to illustrate. In the current example, where five axes are analyzed, we create a five-digit ID code. We opted to assign the digit positions as follows: position 1 = sex, position 2 = race/ethnicity, position 3 = education, position 4 = income, and position 5 = age. For the example stratum mentioned above (“Female, Hispanic, Some college, High income, 30–44 years”), this would result in the five-digit ID code: 23342.

### 2.3 Software and estimation approaches

As noted above, annotated Stata and R code and a simulated instructional data set are provided in the online Supplementary materials. In Stata, standard ‘mixed’ (for linear) and ‘melogit’ (for logistic) commands are used to fit all models using maximum likelihood estimation (MLE). In R we use the lme4 package (functions lmer and glmer) for MLE. There are important pros and cons of using MLE versus Bayesian MCMC approaches for intersectional MAIHDA. MLE, a frequentist approach, is undoubtably more common and therefore more familiar to most researchers. MLE also tends to be faster and to require fewer pre- and post-estimation steps in specifying the model, declaring estimation options, and preparing the results. In our experience with empirical data, MLE also tends to give similar results to MCMC for typical intersectional MAIHDA analyses.

However, MCMC offers an important advantage for intersectional MAIHDA. Namely, using MLE it is not so easy to obtain 95% Confidence Intervals (CI) for many of the key predicted quantities of interest, such as for the interaction effect (on the predicted probability scale) in the logistic model and for the total predicted values in each stratum (in both linear and logistic models, which requires estimating a 95% CI around the sum of fixed and random effects). To obtain *approximate* 95% CI in our MLE demonstration, we have to make certain assumptions, such as there being no sampling covariability between the fixed and random effects, and apply a simulation-based approach. MCMC methods, on the other hand, estimate 95% Credible Intervals for all of the statistics without such assumptions. Therefore, while we illustrate how to obtain approximate 95% CIs using MLE, we encourage researchers to also explore the alternative demonstrations we provide that estimate MAIHDA models using MCMC.

We present additional syntax available for fitting models with MCMC in Stata (using the bayes prefix) and in MLwiN (Browne, 2023; Charlton et al., 2024; Rasbash et al., 2023) (from Stata, using the *runmlwin* command (Leckie & Charlton, 2013)), from: https://doi.org/10.17605/OSF.IO/DTVC3. In the future, additional resources will be made available there as well.

### 2.4 Analysis – linear MAIHDA models

#### 2.4.1 Sample means, grand means, and precision weighted grand means

Before delving into the specifics of model specification, it can be helpful to clarify conceptually the differences between sample means, grand means, and precision weighted grand means, as these are fundamental concepts in multilevel modeling (Raudenbush & Bryk, 2002).

**Fig. 1:**
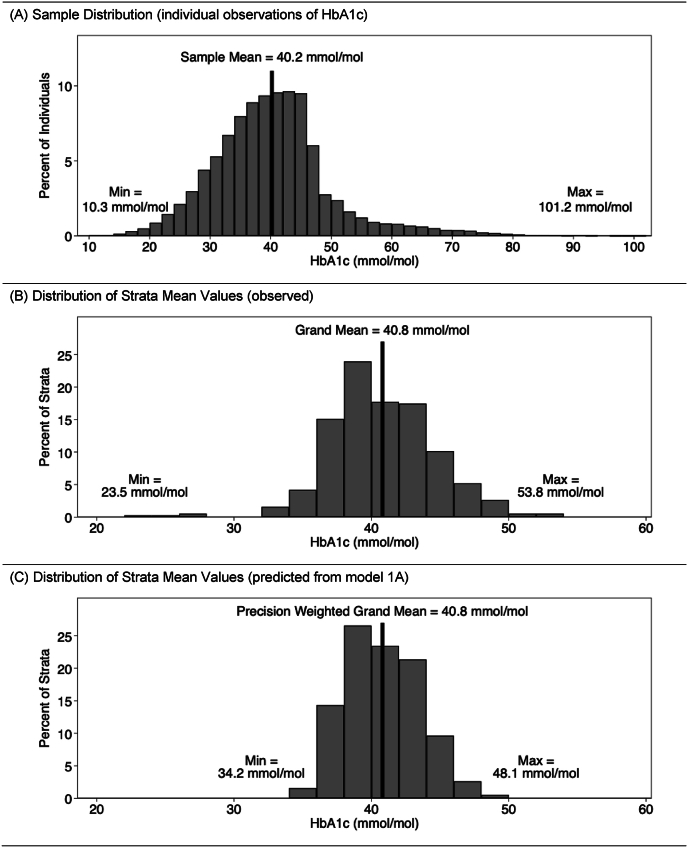
Visualizing distributions – sample mean, grand mean, and precision weighted grand mean.

Consider the simulated data structure: we have N = 33,000 individual observations (level 1) nested in 384 strata (level 2). Each simulated individual has a continuous HbA1c score. The sample distribution of these scores is shown in Fig. 1A. The *sample mean* is simply the mean value of these 33,000 HbA1c scores, which in this case is 40.2 mmol/mol. If we fit a single-level regression with no explanatory variables (i.e., a “null” or empty model with only an intercept), the intercept would estimate this sample mean. Obviously, this would be a complicated way to calculate this statistic, but we note it because it illustrates that this mean makes no reference to stratum groupings or stratum sizes.

If we now calculate the mean HbA1c value in each stratum and plot the distribution of these observed sample means, we obtain the distribution in Fig. 1B. The average of these averages is the *grand mean*, which in this case is 40.8 mmol/mol. As can be seen in this distribution, some strata have very small or large mean values (Min = 23.5 mmol/mol; Max = 53.8 mmol/mol). This could be due to actual underlying disparities in average scores between strata. However, it is likely that some of these extreme values may be reflecting the small sample sizes observed in some strata, and thus these average values will in general be unreliable estimates of their population-statistic counterparts. This in turn will affect the reliability of the grand mean.

In a multilevel linear regression with no explanatory variables the intercept is a *precision weighted grand mean* (PWGM). Similar to the grand mean, the PWGM is an “average of averages,” however, it is now a weighted average of the observed stratum means where the weights are the precision (inverse variance) of the observed stratum means. The predicted stratum means from this model are then reliability adjusted versions of the observed stratum means. Specifically, the observed stratum means are adjusted towards the PWGM as function of their reliability. This produces a “shrunken” distribution (Fig. 1C), whereby the values for smaller strata are shrunk more than the values for larger strata, reflecting their lower reliability. In this model, as discussed elsewhere (Evans et al., 2020), values of the PWGM will lie between the sample mean and grand mean. In this simulated case, the PWGM closely resembles the grand mean, with a value of 40.8 mmol/mol. However, as we see from this distribution, the range of the min/max predicted values are significantly drawn inward after adjustment for strata sample size.

Further descriptive statistics for all three distributions are provided in Table 1. We proceed now to a more formal introduction to the linear MAIHDA models.

#### 2.4.2 Linear models - the simple intersectional model (Model 1A)

Let $y_{ij}$ denote the HbA1c for individual i ($i=1,\ldots ,n_{j}$) in stratum j (j=1,..,J). The simplest intersectional MAIHDA model is the null model. The model, expressed using level-specific equations, can be written as:

Level 1: $y_{ij}=\beta_{0j}+e_{ij}$.
Level 2: $\beta_{0j}=\beta_{0}+u_{j}$.

or equally as a combined equation, where we have substituted the level 2 equation into the level 1 equation, as:

$$y_{ij}=\beta_{0}+u_{j}+e_{ij}$$

where $u_{j}∼N\left(0,\sigma_{u}^{2}\right)$ and $e_{ij}∼N\left(0,\sigma_{e}^{2}\right)$.

In this model, $\beta_{0j}$ is the mean value specific to stratum j, and is decomposed into an overall mean $\beta_{0}$ (the PWGM) and a stratum random effect $u_{j}$. The latter measures how different the mean in stratum j is from the overall mean. The $u_{j}$ are assumed to be normally distributed with mean of 0 and variance $\sigma_{u}^{2}$. The residual $e_{ij}$ measures the deviation of the observed outcome for individual i in stratum j from their stratum mean $\beta_{0j}$ and is also assumed normally distributed with mean 0 and variance $\sigma_{e}^{2}$.

As discussed above, the observed sample means for small strata will be less reliable estimates of their population counterparts than is the case for large strata. Let ${\bar{y}}_{.j}$ denote the observed sample mean in stratum j. The predicted PWGM across all strata ${\hat{\beta }}_{0}$ is a precision weighted average of all 384 values of ${\bar{y}}_{.j}$:

$${\hat{\beta }}_{0}=\frac{\sum_{j=1}^{J}w_{j}{\bar{y}}_{.j}}{\sum_{j=1}^{J}w_{j}},w_{j}=\frac{1}{{\hat{\sigma }}_{u}^{2}+\frac{{\hat{\sigma }}_{e}^{2}}{n_{j}}}$$

where $w_{j}$ is the precision (inverse sampling variance) of ${\bar{y}}_{.j}$.

The predicted mean in each stratum ${\tilde{\beta }}_{0j}$ is then a reliability weighted average of the observed sample mean and the PWGM:

$${\tilde{\beta }}_{0j}={\hat{R}}_{j}{\bar{y}}_{.j}+\left(1-{\hat{R}}_{j}\right){\hat{\beta }}_{0},\;{\hat{R}}_{j}=\frac{{\hat{\sigma }}_{u}^{2}}{{\hat{\sigma }}_{u}^{2}+\frac{{\hat{\sigma }}_{e}^{2}}{n_{j}}}$$

where ${\hat{R}}_{j}$ is the reliability of ${\bar{y}}_{.j}$ as an estimator of $\beta_{0j}$. When $n_{j}$ (the sample size in stratum j) is large, ${\hat{R}}_{j}$ approaches 1 and ${\tilde{\beta }}_{0j}$ tends to the observed stratum mean ${\bar{y}}_{.j}$. However, when $n_{j}$ is small, ${\hat{R}}_{j}$ tends to the VPC (defined below), and ${\tilde{\beta }}_{0j}$ tends towards a VPC weighted average of ${\bar{y}}_{.j}$ and ${\hat{\beta }}_{0}$ with the result that ${\tilde{\beta }}_{0j}$ lies much closer to the PWGM ${\hat{\beta }}_{0}$. Thus, the distribution of ${\tilde{\beta }}_{0j}$ will tend to be shrunk inwards relative to the distribution of ¯y.j towards the PWGM, as shown in Fig. 1C. This means that intersectional MAIDHA will tend to produce more reliable estimates of stratum means when stratum sizes are small than single-level regression approaches that enter a separate fixed effect dummy variable for each stratum (Bell et al., 2019; Evans 2019a; Evans et al., 2018; 2020; Mahendran et al., 2022a).

##### 2.4.2.1 Between-stratum variance and the Variance Partition Coefficient

The null model is an important one in intersectional MAIHDA because of the key statistics it provides summarizing overall inequity in the sample. In addition to generating stratum-specific predictions, it summarizes the degree of outcome heterogeneity within and between strata. The stratum-level variance $\sigma_{u}^{2}$ is a measure that captures the between-stratum inequity, unadjusted for any other factor. The actual practical significance of this value depends partially on how much overall variation there is in the outcome in the sample across individuals. The Variance Partition Coefficient (VPC) provides a measure that takes total individual variation into account. Specifically, the VPC is defined as the proportion of total individual variance in $y_{ij}$ (given here by $\sigma_{u}^{2}+\sigma_{e}^{2}$) that lies between-strata.

$$\text{VPC}=\frac{\sigma_{u}^{2}}{\sigma_{u}^{2}+\sigma_{e}^{2}}$$

The statistic ranges from 0 to 1 and higher values indicate greater practical significance. The VPC is often re-expressed as a percentage by multiplying the initial value by 100. The VPC expresses the contextual influence of the strata for understanding individual inequalities. It also quantifies the intra-stratum correlation or clustering of individual HbA1c within the strata. That is, the correlation in the HbA1c value between two individuals randomly taken from the same stratum. A high VPC would indicate the HbA1c values of individuals from the same stratum are very similar and, simultaneously, very different from the HbA1c of the individuals in other strata. Hypothetically, if the VPC were 100%, then knowing the stratum HbA1c average would tell us the HbA1c of every individual in the stratum. Conversely, if the VPC were 0%, all strata would appear similar to one another and so stratum membership would tell us nothing about the HbA1c of each individual. That is, there is no General Contextual Effect (GCE) of the studied intersectional strata (Merlo, Wagner, Austin, Subramanian, & Leckie, 2018).

#### 2.4.3 Linear models – the additive main effects model (Model 1B)

Sometimes researchers may be interested in estimating interaction effects for strata, or in quantifying the extent to which observed inequities between strata are described by additive versus interaction effects. For this, we fit an additive main effects model (Model 1B) where we enter the set of categorical variables that define the strata as fixed level 2 explanatory variables:

$$y_{ij}=\beta_{0}+\beta_{1}x_{1j}+\cdots +\beta_{p}x_{pj}+u_{j}+e_{ij}$$

$$u_{j}∼N\left(0,\sigma_{u}^{2}\right)$$

$$e_{ij}∼N\left(0,\sigma_{e}^{2}\right)$$

where $x_{1j},\ldots ,x_{pj}$ denote the p dummy variables and $\beta_{1},\ldots ,\beta_{p}$ are their associated regression coefficients needed to specify the categorical variables (in this case, sex, race/ethnicity, education, income and age). In our example, this means entering the five explanatory variables as 12 dummy variables at level 2, for a total of 13 beta regression coefficients including the intercept. Note that, contrary to how these variables may typically be treated in single-level models, here they operate as level 2 variables (and thus are indexed by j, not ij). All other terms are defined as before. The summation $\beta_{0}+\beta_{1}x_{1j}+\cdots +\beta_{p}x_{pj}$ gives the predicted HbA1c score for stratum j based on the additive main effects alone.

Importantly, this model includes no fixed interaction parameters (e.g., we include dummy variables at level 2 for ‘woman’ and ‘Black’ but not for ‘woman × Black’). Instead, the entirety of the interaction effect for stratum j is captured by the stratum random effect $u_{j}$. This term measures the deviation of the stratum means from the values implied by the additive main effects alone. As in Model 1A, the $u_{j}$ are assumed normally distributed with mean 0 and between-stratum variance $\sigma_{u}^{2}$. However, the latter now measures the variance that remains between strata after adjustment for the additive main effects. The residual $e_{ij}$ has the same interpretation as in Model 1A: it is the difference between the observed HbA1c value for individual i in stratum j and what is predicted for stratum j. The residual $e_{ij}$ continues to be assumed normally distributed with mean 0 and within-stratum-between-individual variance $\sigma_{e}^{2}$.

##### 2.4.3.1 An improved way to quantify interactions

The MAIHDA approach has a number of important advantages over conventional single-level regression that include regular interaction effects. The single-level model would take the general form:

$$y_{i}=\beta_{0}+\beta_{1}x_{1i}+\cdots +\beta_{p}x_{pi}+\gamma_{1}x_{1i}x_{2i}+e_{i}$$

$$e_{i}∼N\left(0,\sigma_{e}^{2}\right)$$

where $x_{1i}x_{2i}$ is a two-way interaction between two of the existing dummy variables in the model. Further two- or higher-way interactions may be added as necessary to fully specify all category combinations. Notably, most researchers pay attention only to the predicted value of $y_{i}$ and the estimated regression coefficients, neglecting consideration of variance in favor of comparing averages.

MAIHDA’s advantages over this single-level model can be broadly categorized into theoretical and methodological/practical considerations. From a theoretical perspective, the first advantage comes from differences in model setups: “interaction effects” are predicted for all social strata in MAIHDA, whereas they are only estimated for particular pre-specified combinations of sociodemographic characteristics in the single-level approach.

Second, it is often the multiply privileged strata (such as high-income white men) that are treated as the reference category in analyses in order to investigate interactions for multiply marginalized strata (such as low-income Black women). This has been critiqued as potentially reinforcing the social primacy of multiply advantaged populations by constantly treating them as the default (Choo & Ferree, 2010; Evans et al., 2018). While MAIHDA still specifies references among the fixed parameters, it matters less which categories are used as reference in the fixed part of the model because they are treated collectively (by calculating $\beta_{0}+\beta_{1}x_{1j}+\cdots +\beta_{p}x_{pj}$) as additive reference levels for all strata.

Third, the single-level approach encourages consideration of individual regression coefficients $\beta_{1},\ldots ,\beta_{p}$ rather than the collective additive effect ($\beta_{0}+\beta_{1}x_{1j}+\cdots +\beta_{p}x_{pj}$). In practice, this seems to result in reversion to single-axis thinking about inequity (e.g., asking whether the effect of race(ism) is more important than income inequality) when interpreting model results, which is counter to the stated purpose of intersectional comparisons.

There are also important methodological and practical advantages to MAIHDA. First, as additional categories of inequality, marginalization, or identity are added to intersectional analyses, the number of regression coefficients in single-level models grows geometrically, while the number in MAIHDA models grows linearly. For instance, in this simulated example, we could fit a single-level model by including all of the additive main effects and all first-, second-, third-, and higher-order interactions between them, for a total of 384 regression coefficients. Meanwhile, MAIHDA only includes 13 regression coefficients in $\beta_{0}+\beta_{1}x_{1j}+\cdots +\beta_{p}x_{pj}$ and a random effect variance $\sigma_{u}^{2}$ to summarize the distribution of 384 stratum interaction effects. This makes MAIHDA models more parsimonious, and therefore more scalable for handling high-dimensional interactions.

Second, the relative parsimony of MAIHDA means that it is easier to interpret results. There are far fewer regression coefficients, and the interpretations of each of these is more intuitive. The additional variance parameter at level 2 in MAIHDA is also a useful statistic for quantifying between-stratum inequities, and there is no comparative statistic obtained in the single-level model.

Finally, as discussed previously, MAIHDA’s precision weighting facilitates more reliable prediction for strata with smaller sample sizes. Knowing this, a researcher using a data set with a given sample size may have more leeway to include additional categories (e.g., sexuality, disability) in their analysis with reduced (though not entirely eliminated) concern for small sample problems. While multiple testing problems are always of potential concern, with MAIHDA these concerns are partially overcome, compared with single-level approaches, thanks to the shrinkage of the stratum random effects in the model (Bell et al., 2019).

##### 2.4.3.2 The between-stratum variance attributable to interaction effects

Because of the shifted definition of the between-stratum variance $\sigma_{u}^{2}$ in Model 1B, the interpretation of the VPC in this model is also slightly different. The VPC now represents the proportion of the total variance that remains (after adjustment for additive effects) that is attributable to interaction effects. By 'attributable to interaction effects' we mean that some portion of the between-stratum variance (or inequalities) are not adequately described with consisent, additive patterns. Generally, we will expect a considerable percent of the between-stratum variance observed in Model 1A to be explained by inclusion of additive effects, and thus the between-stratum variance and VPC will both be considerably reduced in Model 1B.

To quantify the extent to which the between-stratum variance reduces between Models 1A and 1B, we can calculate the Proportional Change in Variance (PCV):

$$\text{PCV}=\frac{\sigma_{u\left(\text{Model}\;A\right)\;}^{2}-\sigma_{u\left(\text{Model}\;B\right)\;}^{2}}{\sigma_{u\left(\text{Model}\;A\right)\;}^{2}}$$

The PCV is interpreted as the proportion of the total between-stratum variance (in the simple intersectional model) that is *explained* by adjustment for the additive main effects (in the intersectional main effect and interactions model). The complement of this value 1−PCV quantifies the between-stratum variance that is unexplained by adjustment for additive effects and therefore attributable to interaction effects. The PCV is often multiplied by 100 to express it as a percentage. When the PCV value is meaningfully less than 100%, this indicates interaction effects are necessary to accurately characterize observed inequities between strata.

### 2.5 Analysis – logistic MAIHDA models

#### 2.5.1 Predicted probabilities

Fitting logistic regression versions of MAIHDA models is similar to the linear case, but with some important differences. In this section, we focus on those differences. To begin with, the outcome $y_{ij}$ is now a binary measure (diabetic: 1 = yes, 0 = no) constructed by dichotomizing individuals' continuous HbA1c scores, where ≥48 mmol/mol is indicative of a ‘diabetic range.’ Thus, rather than modelling the mean value of HbA1c in each stratum, we estimate the probability of being in the diabetic range in each stratum. For simplicity, we will often say the probability of being diabetic (or the probability of having diabetes).

The simple null MAIDHA model (Model 2A) for this binary outcome is written as the following multilevel logistic regression model:

$$y_{ij}∼\text{Bernoulli}\left(\pi_{j}\right)$$

$$\text{logit}\left(\pi_{j}\right)\equiv \log\left(\frac{\pi_{j}}{1-\pi_{j}}\right)=\beta_{0}+u_{j}$$

$$u_{j}∼N\left(0,\sigma_{u}^{2}\right)$$

where $y_{ij}∼\text{Bernoulli}\left(\pi_{j}\right)$ states that $y_{ij}$ is modelled as following the Bernoulli distribution with probability $\pi_{j}$, logit(∙) denotes the logit link function which maps these probabilities onto the logit scale, and so $\text{logit}\left(\pi_{j}\right)$ denote the logit of the probability of diabetes, $\log\left(\frac{\pi_{j}}{1-\pi_{j}}\right)$ denotes the log-odds of diabetes, and $\frac{\pi_{j}}{1-\pi_{j}}$ denotes the odds of diabetes. Note that on the right hand side of the equation there is no individual-level residual. This is because the model equation is expressed in terms of the expected outcome $\pi_{j}$ rather than the observed outcome $y_{ij}$.

The additive main effects version of the logistic MAIHDA model is the same as the null version, except that we now include the additive main effects in the fixed part of the model, just as we did for the linear Model 1B. Thus, Model 2B is:

$$y_{ij}∼\text{Bernoulli}\left(\pi_{j}\right)$$

$$\text{logit}\left(\pi_{j}\right)\equiv \log\left(\frac{\pi_{j}}{1-\pi_{j}}\right)=\beta_{0}+\beta_{1}x_{1j}+\cdots +\beta_{p}x_{pj}+u_{j}$$

$$u_{j}∼N\left(0,\sigma_{u}^{2}\right)$$

Importantly, while in linear models the additive main effects and interaction effects are estimated directly on the outcome scale, in logistic models they are not. The logit link function means that these terms are estimated on the logit or log-odds scale, while it is the probability or risk scale which is typically preferred for interpretability reasons, particularly by clinicians (Grimes & Schulz, 2008; Sackett, Deeks, & Altman, 1996). This makes it more complex to interpret the results without first converting these back to the probability scale. For instance, we can no longer directly interpret $u_{j}$ as the change in mean outcomes (i.e., shift in probabilities) attributable to interaction effects.

While it is interesting to examine the odds ratios (ORs, exponentiated regression coefficients) associated with the dummy variables, we will generally be more interested in our intersectional analysis in calculating three things: (1) the *total predicted probability in each stratum*, calculated by $\pi_{j}={\text{logit}}^{-1}\left(\beta_{0}+\beta_{1}x_{1j}+\cdots +\beta_{p}x_{pj}+u_{j}\right)$ where ${\text{logit}}^{-1}\left(∙\right)$ denotes the inverse of the logit function. (2) What the predicted probability would be if we only considered additive main effects, calculated by $\pi_{j}^{A}={\text{logit}}^{-1}\left(\beta_{0}+\beta_{1}x_{1j}+\cdots +\beta_{p}x_{pj}\right)$. And (3), the difference in the predicted probability between the total probability and that based only on additive main effects $\pi_{j}^{B}=\pi_{j}-\pi_{j}^{A}$. The latter is interpreted as that part of the predicted probability attributable to interaction effects.

#### 2.5.2 The VPC, AUC and PCV in logistic models

In logistic regression models, we do not estimate the individual-level variance term $\sigma_{e}^{2}$ that is needed to calculate the VPC. Various methods have been proposed for calculating the VPC in such models (Goldstein, Browne, & Rasbash, 2002), the most widely applied of which is the latent response approach. This involves setting $\sigma_{e}^{2}$ equal to the variance of the standard logistic distribution $\frac{\pi^{2}}{3}\approx 3.29$ where π here denotes the mathematical constant 3.142. The equation for VPC is then the same as before, except with $\sigma_{e}^{2}=3.29$.

The interpretation of the VPC is substantively similar as in the case of the linear Model 1A. However, in logistic regression we can also use the predicted probability to calculate area under the receiver operating characteristic curve (AUC) statistic.

The AUC is another measure of discriminatory accuracy, and as such it measures the accuracy of knowing the intersectional stratum of an individual for discriminating individuals with diabetes from individuals without diabetes. Formally, the AUC can be defined as the probability that a randomly selected individual with diabetes will have a higher predicted probability than a randomly selected individual without diabetes. The AUC takes a value between 0.5 and 1.0 (or 50% and 100%) where 0.5 corresponds to the intersectional strata having no discriminatory accuracy and 1.0 corresponds to perfect discriminatory accuracy. In our study, the predicted probability is only dependent on the intersectional stratum and so the AUC is simply the probability that a randomly selected individual with diabetes belongs to an intersectional stratum with a higher prevalence of diabetes than does a randomly selected individual without diabetes. Given this, we would not expect to find particularly high AUC scores, as we wouldn’t expect our model to be particularly good at diagnosing diabetes at an individual level. As such we avoid quantifying AUC values according to traditional “good/bad” thresholds of predictive accuracy, as this is not the intended use of this statistic in this analysis. In the null model, when the VPC equals 0% the AUC equals 50%, while when the VPC equals 100% the AUC also equals 100% (Merlo, Wagner, & Leckie, 2019).

The PCV is calculated in the same way for logistic models as for linear models, as the proportional reduction in the between-stratum variance (on the logit or log-odds scale) as we move from Model 2A to Model 2B.

#### 2.5.3 A computationally more efficient approach

It can be computationally slow to estimate the above logistic regression models when data are large, particularly if Bayesian MCMC methods are used. Given that the only explanatory variables are defined at the stratum level, an alternative and computationally more efficient approach is to collapse the data down to one record per stratum and to fit equivalent models to *binomial counts* of the number of individuals with diabetes in each stratum. In our application, the data would reduce from 33,000 records, one for each individual, to 384 records, one for each stratum, with no loss of information. The response distribution becomes $y_{+j}∼\text{Binomial}\left(n_{j},\pi_{j}\right)$ where $y_{+j}$ denotes the number of individuals with diabetes in stratum j. This approach leads to equivalent models, with identical parameter estimates and model predictions as we move from the binary to binomial case. However, the binomial version is far faster to estimate due to the smaller dataset size. In the MCMC versions of the code available online, we estimate logistic models using only the binomial version of the outcome.

## 3 Presenting results from intersectional MAIHDA

### 3.1 Presenting and interpreting tables of results

We have already discussed the descriptive statistics presented in Table 1, Table 2, and we encourage such information to be included in MAIHDA publications. Notably, it can be useful to provide information on the sample size and its distribution across individual characteristics (e.g., gender, race/ethnicity) as well as across the strata (e.g., how many and what percent of strata are of a given sample size or larger). We suggest including, if possible, a Supplemental Table online that lists all analyzed strata, their sample sizes, and key results (e.g., the observed and predicted stratum means and the decomposition of the later into the parts attributed to additive main effects vs. interactions). Also included in our Table 1, are descriptive statistics showing the differences between the sample mean, grand mean, and precision-weighted grand mean. Such information may not be necessary in most publications, but it is advisable to include standard descriptive statistics related to the dependent variable or other key variables.

Including the results of models, such as those shown in Table 3, is essential. Here, we present the null (Models 1A and 2A) and additive main effects (Models 1B and 2B) results for our continuous and binary outcome models obtained using MLE in Stata. (Unless otherwise noted, all figures and tables in the manuscript were created from Stata results, however the equivalent syntax is available in the provided R code.) Regression coefficients are presented where appropriate, and within- and between-stratum variances are included. We also present the VPC and PCV, and the AUC in logistic models.

**Table 3:** Parameter estimates for linear models of HbA1c (mmol/mol) and logistic models of diabetes (HbA1c ≥ 48 mmol/mol).

	Linear Model 1A		Linear Model 1B		Logistic Model 2A		Logistic Model 2B	
	Estimate	[95% CI]	Estimate	[95% CI]	OR	[95% CI]	OR	[95% CI]
**Fixed Effects: Regression Coefficients**								
Intercept	40.79	[40.44, 41.13]	38.03	[37.45, 38.62]	0.16	[0.15, 0.17]	0.09	[0.07, 0.10]
Sex								
Male (Ref)			–				–	
Female			−0.52	[-0.83, −0.21]			0.90	[0.83, 0.98]
Race/Ethnicity								
White Non-Hispanic (Ref)			–				–	
Black Non-Hispanic			4.45	[4.08, 4.82]			2.36	[2.15, 2.59]
Hispanic/Latino			1.04	[0.66, 1.42]			1.19	[1.07, 1.32]
Education								
Less than high school (Ref)			–				–	
Completed high school			−0.54	[-1.01, −0.07]			0.94	[0.83, 1.06]
Some college no degree			−0.55	[-1.03, −0.06]			0.89	[0.79, 1.02]
College degree or more			−1.13	[-1.61, −0.65]			0.81	[0.71, 0.92]
Income (% Poverty Threshold)								
Low (<100%) (Ref)			–				–	
Low-middle (100%–199%)			0.18	[-0.23, 0.60]			1.09	[0.98, 1.22]
High-middle (200%–399%)			−0.30	[-0.73, 0.12]			0.96	[0.86, 1.08]
High (400% or more)			−1.33	[-1.82, −0.83]			0.79	[0.69, 0.91]
Age								
18–29 years (Ref)			–				–	
30–44 years			1.10	[0.65, 1.56]			1.35	[1.18, 1.55]
45–59 years			1.81	[1.35, 2.27]			1.55	[1.35, 1.77]
60+ years			5.50	[5.03, 5.98]			3.06	[2.68, 3.49]
**Random Effects: Variances**								
Stratum-Level	9.33	[7.82, 11.14]	0.80	[0.55, 1.15]	0.39	[0.32, 0.49]	0.03	[0.02, 0.06]
Individual-Level	90.26	[88.89, 91.66]	90.27	[88.89, 91.66]	–			
**Summary Statistics**								
Variance Partition Coefficient (VPC)	9.4%		0.9%		10.7%		0.9%	
Proportional Change in Variance (PCV)			91.4%				92.2%	
Area Under Receiver Operating Characteristic Curve (AUC)					0.68		0.67	

*Notes:* MLE estimation used for all models shown. All values are derived from simulated data and are for demonstration purposes only. 95% CIs shown in parentheses. VPC for logistic models are calculated using the latent response approach ($\sigma_{e}^{2}$ is set equal to the variance of the standard logistic distribution $\frac{\pi^{2}}{3}\approx 3.29$ where π here denotes the mathematical constant 3.14159).

The intercept in the null linear Model 1A is interpreted as the precision-weighted grand mean (that is, overall average value) of HbA1c in the simulated sample. In this model, we see a VPC = 9.4%, which indicates a relatively large amount of clustering at the stratum-level. A similar result, as expected, is observed in the logistic version of the model (VPC = 10.7%). As a point of reference, most studies using multilevel models to examine individuals (level 1) nested in neighborhoods, schools, or workplaces (level 2) will often see VPCs less than 5%, and they rarely exceed 10% (Subramanian, & James O’Malley, 2010). Another way to interpret this VPC is that there are considerable inequities in HbA1c scores across intersectional strata. It is important to keep in mind, however, that the VPC is a reflection of both inequity between strata and *also* individual level variance. Thus, if there is considerable person-to-person variation in an outcome, then even meaningful inequities between strata can be obscured in the VPC. It is therefore also important to examine the actual range of predicted values across strata and to present visualizations of these inequities (see below) when characterizing inequity.

When describing results such as those in linear Model 1B, readers may benefit from a reminder that the fixed effect values presented for variables like gender and race/ethnicity are merely the additive—that is, *overall*—pattern predicted by the model. For instance, in these data, the average female stratum has an HbA1c score that is 0.52 mmol/mol lower than the average male stratum, holding all other variables constant. Black and Hispanic/Latino strata typically have higher HbA1c scores by 4.45 mmol/mol and 1.04 mmol/mol, respectively. However, these additive patterns can obscure key results that are seen best when examining the intersections (total predicted value for each stratum and how this decomposes into that part due to additive main effects and that part due to interactions).

There are many ways to inspect patterns of results that are inclusive of interaction effects. One simple method is to generate lists of the strata with the highest and lowest predicted values (as in Table 4). In these results, for instance, it is generally the case that women have lower probabilities of HbA1c scores in the diabetic range, however the stratum with the highest predicted percentage with diabetes (stratum 22124, 40.5% diabetic) is Black women with less than high school education, low-middle income, and age 60+ years. Another method is to plot the final predicted values for all strata, perhaps organized by low-to-high rank or a qualitative grouping of interest (we address results visualization in the following sections).

**Table 4:** Inspection of five highest and lowest ranked strata for predicted mean HbA1c (Model 1B) and percentage diabetic (model 2B).

Predicted Mean HbA1c (Model 1B)									
Rank	Stratum	Sex	Race	Education	Income	Age	n	Predicted Mean HbA1c	Approximate 95% CI
*5 Lowest*									
1	11441	Male	White	College plus	High	18–29	111	34.7	[33.4, 36.1]
2	21341	Female	White	Some college	High	18–29	43	35.0	[33.4, 36.6]
3	21331	Female	White	Some college	Mid-high	18–29	136	35.1	[33.8, 36.4]
4	21241	Female	White	High school	High	18–29	15	35.4	[33.7, 37.2]
5	21441	Female	White	College plus	High	18–29	114	35.7	[34.3, 37.0]
*5 Highest*									
380	12324	Male	Black	Some college	Low-mid	60+	33	47.6	[46.0, 49.3]
381	12134	Male	Black	< High school	Mid-high	60+	29	47.7	[46.0, 49.3]
382	22214	Female	Black	High school	Low	60+	157	47.8	[46.5, 49.0]
383	22124	Female	Black	< High school	Low-mid	60+	114	48.1	[46.8, 49.5]
384	12114	Male	Black	< High school	Low	60+	112	48.4	[47.0, 49.7]

Predicted Percentage Diabetic (Model 2B)									
Rank	Stratum	Sex	Race	Education	Income	Age	n	Predicted % Diabetic	Approximate 95% CI
*5 Lowest*									
1	21441	Female	White	College plus	High	18–29	114	4.9	[3.4, 6.8]
2	11441	Male	White	College plus	High	18–29	111	4.9	[3.5, 6.9]
3	21341	Female	White	Some college	High	18–29	43	5.1	[3.6, 7.3]
4	21241	Female	White	High school	High	18–29	15	5.4	[3.8, 7.7]
5	21442	Female	White	College plus	High	30–44	445	5.6	[4.3, 7.4]
*5 Highest*									
380	12224	Male	Black	High school	Low-mid	60+	44	37.5	[30.2, 45.4]
381	12134	Male	Black	< High school	Mid-high	60+	29	37.7	[30.0, 46.1]
382	12324	Male	Black	Some college	Low-mid	60+	33	37.8	[30.2, 46.0]
383	12114	Male	Black	< High school	Low	60+	112	38.9	[32.3, 46.0]
384	22124	Female	Black	< High school	Low-mid	60+	114	40.5	[33.8, 47.5]

*Notes*: 95% CI are approximate only because the model assumes no sampling covariability between the regression coefficients and the stratum random effects.

As expected, the VPC in both of the additive main effects models has decreased from the null model values, from 9.4% to 0.9% in the linear case and from 10.7% to 0.9% in the logistic case. These non-zero values indicate that some stratum inequity is left unexplained by additive main effects. A different way to measure this is the PCV. The PCV in both cases is >90%, indicating that this percent of the total variance between strata is accounted for by the contributions of additive main effects—with the rest attributable to interactions effects.

Finally, we calculate the AUC for the logistic models: 0.68 and 0.67 for Models 2A and 2B respectively. Whilst this suggests the model is not particularly good at predicting whether an individual is diabetic, that is not surprising; in a model that only contains variables relating to individuals’ broad identity characteristics, we would not expect to find a higher level of discriminatory accuracy. That the AUC statistics are effectively the same for Models 2A and 2B is also not surprising as moving from one model to the next we only added the stratum definitional characteristics to the model which already had stratum random effects and so this will make little difference to the predicted stratum means and therefore the accuracy of the model to predict individual outcomes.

Given the large number of estimates generated by MAIHDA, and the many possible analysis permutations the approach opens up, it can be easy to lose focus on the key research questions of interest. We recommend, while planning a set of analyses, periodically reminding oneself of the research questions and aims. For instance, common questions might include ‘what is the degree of inequality across strata on an outcome?’ Focusing on the magnitude and distribution of predicted stratum values and on the VPC from the null model (with reference to the within and between variances), are typical ways to approach this question. Researchers may wish to highlight results for particular strata of interest, or to make specific between-group comparisons, in which case the general results (such as VPC and PCV) can be provided in tables but emphasized less in the manuscript text.

### 3.2 Visualizing results – ranked predicted values

**Fig. 2:**
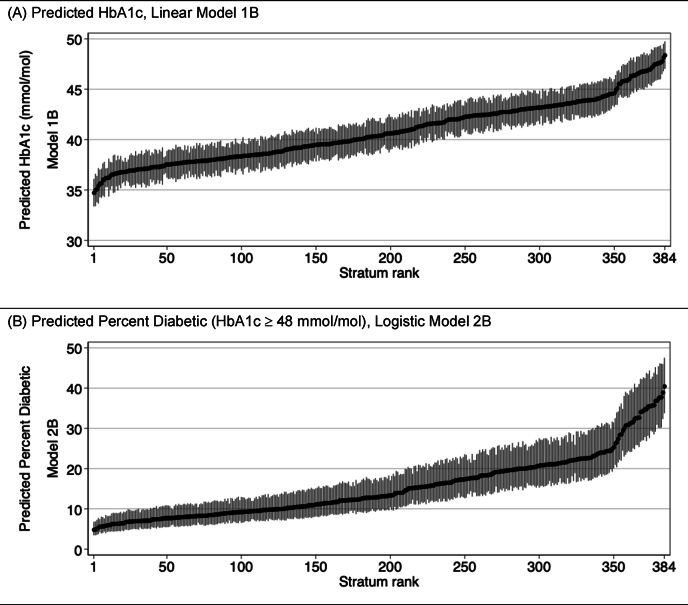
Predicted values by stratum for all 384 strata, ranked low to high. *Notes*: All values are derived from simulated data and are for demonstration purposes only. Markers indicate predicted value for each stratum. Spikes indicate approximate 95% CIs.

While tables of predictions for each stratum can be presented in the manuscript body or online supplements, as desired, we encourage the use of visualizations for these results, such as those provided in Fig. 2. Such figures often illustrate the range, spread, and pattern of inequities between strata better than the numeric values. In Fig. 2 we opted to present the predicted values for the 384 strata ranked from low to high, without attempting to label each marker with a stratum ID, as this would have made the figure nearly illegible. For analyses with fewer strata, labeling with strata ID may be desirable, and we will show such an example shortly.

An alternative way to present such a figure, not show here, would be to organize the plots so stratum predictions are grouped by labels, such as man/woman, white/Black/Hispanic (Holman et al., 2020; Silva & Evans, 2020). This can be a useful way to detect important patterns. In analyses where there are many strata, however, this labeling and organization can become cumbersome. Alternatives might involve plotting only a subset of strata in a single figure, or reversing the axes (so predicted values of the outcome are plotted on the X-axis and strata labels are given on the Y-axis).

### 3.3 Visualizing results – ranked residuals

**Fig. 3:**
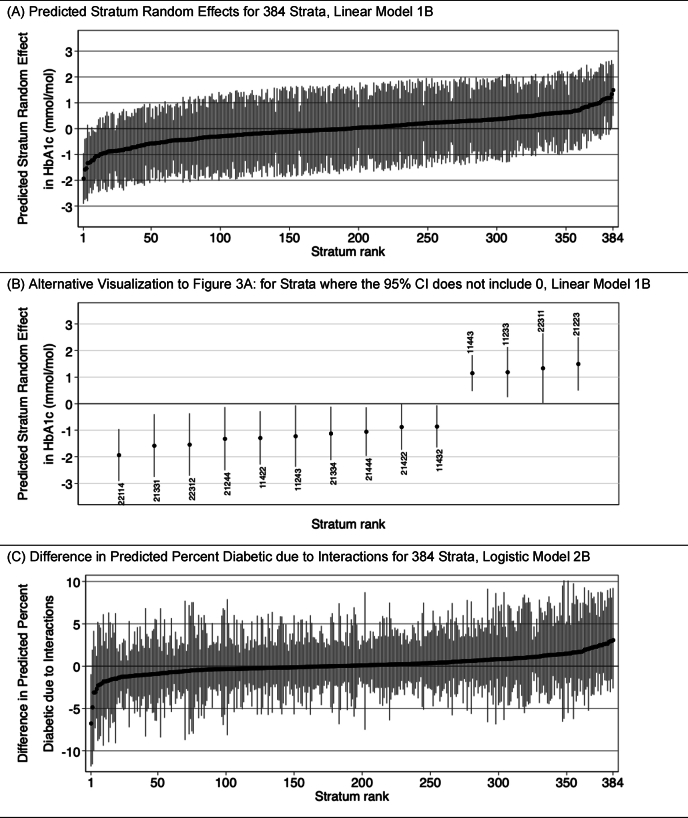
Predicted stratum interaction effects, ranked low to high. *Notes*: All values are derived from simulated data and are for demonstration purposes only. Markers indicate predicted value for each stratum. Spikes indicate approximate 95% CIs. Fig. 3B: Five-digit stratum ID numbers reflect coding as follows: *Digit 1* = sex [1 = Male; 2 = Female]. *Digit 2* = race/ethnicity [1 = white non-Hispanic; 2 = Black non-Hispanic; 3 = Hispanic or Latinx]. *Digit 3* = education [1 = Less than high school; 2 = Completed high school; 3 = Some college no degree; 4 = College degree or more]. *Digit 4* = income[1 = Low income; 2 = Low-middle income, 3 = High-middle income; 4 = High income]. *Digit 5* = age[1 = age 18–29 years; 2 = age 30–44 years; 3 = age 45–59 years; 4 = age 60+ years].

Another useful visualization involves plotting predicted stratum random effects: the $u_{j}$ from Model 1B in the linear case (Fig. 3A), or the difference in the predicted probability between the total predicted probability in stratum j and the probability based on additive main effects $\pi_{j}^{B}=\pi_{j}-\pi_{j}^{A}$ in the logistic case (Fig. 3C). An alternative version of the plot is given in Fig. 3B, which is the same as Fig. 3A but only shows markers for where the 95% confidence interval around the estimate of $u_{j}$ does not encompass 0, a rough measure of statistical significance (though we give the usual caveats for the use of significance testing, and also (re)emphasize that these are only approximate 95% CI). Here stratum ID codes are shown to facilitate interpretation. An important reminder, as we examine and interpret these values, is that a large residual interaction effect does not necessarily mean that the stratum will be more advantaged/disadvantaged in absolute terms (inclusive of additive and interaction effects), but rather it means the final predicted value for the stratum deviates meaningfully from what is predicted based only on the additive effects.

## 4 Discussion

In this section, we address remaining big-picture issues in quantitative intersectional analysis using MAIHDA: (1) What are the fundamental goals for quantitative intersectional approaches and how does MAIHDA address these goals?; (2) How should researchers interpret situations where inequalities are found to be well described by additive (as opposed to additive + interaction) parameters?; and finally, (3) What are extensions and future directions for the MAIHDA approach?

### 4.1 Fundamental analytic goals for quantitative intersectional approaches

Evans, Leckie and Merlo (2020) identify three fundamental analytic goals for quantitative intersectional analyses: (1) mapping averages to identify inequities across strata; (2) quantifying heterogeneity of outcomes within and between strata, both to understand variability/inequality and to gauge the discriminatory accuracy of the strata categorizations used; and (3) to estimate interaction effects.

#### 4.1.1 Fundamental goal #1: mapping inequity in terms of average differences

Mapping predicted means (or probabilities) of outcomes across intersectional strata is, at its heart, a descriptive exercise intended to quantify the magnitude of inequities and to identify strata that are particularly high (or low) risk. When considered alongside information from the other fundamental goals, this can inform public policy to address underlying drivers of the inequity.

It is important to note that if MAIHDA is being used to predict the mean outcome in each stratum, then this can be accomplished using either the null or additive main effects models. In theory, the value of $\beta_{0}+u_{j}$ from the null model should be equal to the value of $\beta_{0}+\beta_{1}x_{1j}+\cdots +\beta_{p}x_{pj}+u_{j}$ from the main effects model. However, in practice, one typically sees some minor differences in the final predictions for smaller strata between the two approaches, due to the different way shrinkage plays out. Though it may be marginally easier to compute final predictions for strata using the null model, it is preferable to produce predictions using the main effects model (though rigorous testing of this remains to be done in future simulation studies). The reason is that predictions (particularly for small strata) will, in the null model, be shrunk toward a single value that is the same for all strata (the PWGM, given by the intercept). In contrast, in the main effects model, the predictions will be shrunk toward stratum-specific additive combinations of the relevant main effects regression coefficients ($\beta_{0}+\beta_{1}x_{1j}+\cdots +\beta_{p}x_{pj}$). These strata-specific predictions from the main effects model will likely be closer to the true stratum means of interest than the single common prediction from the null model. It is therefore more appropriate to shrink towards these strata-specific predictions.

In principle, conventional single-level regression with interaction effects are also capable of making stratum-specific predictions. However, as we have discussed, MAIHDA has a number of key advantages that make it a stronger choice in many situations, including: (1) providing precision-weighted stratum means and thus more reliable predictions when sample sizes are small, (2) allowing more scalability to include additional axes of marginalization without significant loss of model parsimony, and (3) ease of interpretation.

In addition to the methodological and theoretical advantages previously mentioned, MAIHDA approaches the modeling of intersections by treating them as *contexts* within which individuals are nested, analogous to physical environments such as schools or neighborhoods in multilevel models. This is ultimately a more satisfying representation of intersections: axes of marginalization come together to form unique positions in a social landscape, the axes are disentangleable, and they cannot be understood in isolation.

Predicting stratum means (or probabilities) is a useful starting point for quantifying inequities and identifying disproportionately affected groups. However, an exclusive focus on the analysis of differences between group averages can be misleading (see literature on the “tyranny of the averages” (Merlo, 2014, 2018; Merlo & Wagner, 2013)). The first goal alone is therefore insufficient because it ignores within- and between-stratum heterogeneity. Goals 2 and 3 are needed to fully characterize inequities from an intersectional perspective. It is necessary to interpret the specific differences between strata averages values together with the size of the VPC and the discriminatory accuracy of the strata.

#### 4.1.2 Fundamental goal #2: mapping inequity in terms of variances and heterogeneity

Beyond the theoretical and practical advantages of MAIHDA, it is in the second goal of quantitative intersectional analysis where the major differences between single-level and MAIHDA approaches becomes apparent. Single-level approaches do not typically focus on measures of heterogeneity and discriminatory accuracy. In intersectional MAIHDA there are a variety of statistics available to describe variation *within* and *between* strata, including individual-level and stratum-level variance parameters $\sigma_{e}^{2}$ and $\sigma_{u}^{2}$, the VPC, and the PCV.

In addition to quantifying the magnitude of inequities between strata relative to total variation in the sample, the VPC also serves as a measure of discriminatory accuracy. *Discriminatory accuracy* refers to the predictive ability of a characteristic to sort cases from non-cases (e.g., to tell who will have HbA1c in the diabetic range) or to tell how correlated outcomes are expected to be. For logistic models, the AUC serves a similar function. Today, few social, medical, and public health scientists would argue that knowing someone’s gender, race, or socioeconomic status alone would be sufficient to predict the outcome for that individual with any degree of accuracy, as for most outcomes there is considerable heterogeneity of outcomes *between* individuals even with the same characteristics. Still, there is a tacit attitude promoting the idea that observed stratum inequities are always meaningful, at least if they are statistically “significant.”

Since sociodemographic characteristics are at best a rough proxy for a wide range of social experiences that may cluster at the group level and which, in turn, are implicated in complex causal pathways that lead to health outcomes, we do not generally expect the VPC (or the AUC) to be exceptionally large or to have very strong discriminatory accuracy. This is not indicative of a problem, per se. Rather, thinking in terms of discriminatory accuracy provides a valuable check against over-interpreting between-stratum differences or making assumptions that stratum labels determine individual outcomes. For example, past research that has noted racial/ethnic inequities in health have led to over-interpretation and labeling of racial minorities as “high risk”—this can obscure the fact that many non-minorities also suffer from adverse health outcomes and vice versa and it can run the risk of stigmatizing the labeled populations. Furthermore, the attachment of a “high risk” label can lead to misattribution of causation where it is group membership itself that is blamed for poor outcomes, rather than the discrimination, marginalization, or other social experiences associated with group membership.

A low VPC should not be interpreted (in isolation and separate from considering group average differences) as indicating low levels of inequity. Consider a recent substantive example where an intersectional MAIHDA study of birthweight found typical, modest VPCs (VPC = 2.9% among singletons and 3.1% among twins) (Evans, Nieves, Erickson, & Borrell, 2023). However, it would be misleading to interpret this as indicating low levels of inequity between strata. There are many reasons why birthweight varies and this is reflected in a high individual-level variance $\sigma_{e}^{2}$, reducing the VPC score. The significance of the between-stratum variance was apparent when examining the average scores: the difference in predicted mean birthweights for the highest and lowest scoring strata was 388 g for singletons and 435 g for twins, which is a clinically meaningful difference.

MAIHDA fits well with the concept of proportionate universalism, discussed by Marmot, regarding resource allocation in public health (Marmot, 2014). That is, policy responses to health inequities should be inclusive across the health gradient, but with a scale and intensity that is proportionate to the level of disadvantage that caused the health inequities. Considering both between- and within-stratum variability alongside average differences provides a more complete picture of inequities, which can better inform resource allocation and public policy decisions.

#### 4.1.3 Fundamental goal #3: measuring inequities in terms of interaction effects

The third fundamental goal of quantitative intersectional analysis is to estimate interaction effects between axes of marginalization for all points of intersection. Broadly, the purpose of including interaction effects in conventional and MAIHDA models is to allow predicted outcomes to be unique in each stratum, or stated differently, for the association between one axis (gender) and the outcome to be different for different race/ethnicities (e.g., Black verses white). In conventional models, these interactions are regression coefficients and are evaluated for statistical significance. However, this setup means that only some combinations of sociodemographic characteristics have interaction effects associated with them.

In MAIHDA, in the main effects model, every stratum analyzed has an interaction effect given by $u_{j}$ which captures the extent to which the final predicted value for stratum j (given by $\beta_{0}+\beta_{1}x_{1j}+\cdots +\beta_{p}x_{pj}+u_{j}$) differs from what would be expected based purely on additive main effects ($\beta_{0}+\beta_{1}x_{1j}+\cdots +\beta_{p}x_{pj}$). This can help in the identification of unusual breaks with additive inequity patterns, challenging dominant narratives or understandings of health issues, and bringing attention to the needs of often-invisibilized populations. However, as with the other goals, estimating interactions alone is insufficient for a complete understanding of inequities. Only together do these three goals provide a comprehensive quantitative intersectional analysis.

### 4.2 What if inequities are mostly additive?

Of curiosity to many researchers is the following situation: the VPC and between-stratum variance $\sigma_{u}^{2}$ in the null model suggest inequities exist (e.g., VPC = 10%), but in the additive main effects model (Model 1B or 2B) the VPC has shrunk to nearly 0% and the PCV is close to 100%. It might be tempting to conclude that inequities between strata are entirely accounted for by additive effects, and there are no interactions. This may well be the case, however, there are a few caveats to consider.

First, the VPC and PCV are summary measures characterizing patterns in the sample of strata as a whole. Imagine, for instance, we analyze 384 strata and all but one are well explained by additive main effects. The remaining stratum, on the other hand, has a significant interaction effect. This important interaction would be watered down and obscured by only looking at the summary measures of VPC and PCV. Such standout interactions will be better spotted by looking for individual strata with significant interaction effects (e.g., Fig. 3B).

Second, MAIHDA is expected to provide more conservative, shrunken estimates than conventional approaches. It is possible that meaningful interactions do exist in the population, but they may not be visible in the sample due to insufficient sample size to detect small effects. In other words, MAIHDA helps to protect against Type I (false-positive) errors by using shrinkage to pull effects toward null/zero, and this is generally a valued property of the approach, however it also means that even true interaction effects may not be detected if strata are too small.

Finally, what happens if there is truly no interaction? That is, the between-stratum variance $\sigma_{u}^{2}$ is not significant in the additive main effects model. Has this “disproved intersectionality”? The answer is no—this is not a problem, nor is it disproving intersectionality. It is also still worth conducting assessments of inequity using an intersectional framework. The reason for this is fairly simple to understand if we recall the distinction between *experiences* within interlocking systems of oppression (exposures) and *interaction effects* (outcomes) (Evans, 2019a; Evans & Erickson, 2019).

Employed in quantitative analysis, we are using the intersectional framework to inform our decisions for what to study (e.g., gender/sexism, race(ism), ableism), what comparisons to make and what inequities to search for, and how to interpret our results. Finding statistically significant interaction effects simply means that those experiences left an imprint on a *particular outcome* in a *particular sample* at a *particular point in time* that required interactions as well as additive effects to characterize them.

Finding *no* evidence of interaction effects, on the other hand, does not mean systems of oppression are not interlocking, nor that they are unimportant, nor that they don’t form unique social experiences at different intersections. The use of the intersectional framework for inequity estimation remains theoretically sound, allows for the possibility of particular populations to be especially harmed above and beyond expectations, and ultimately the totality of those inequities is the main point of the analysis. It comes back to being critical of the systems that gave rise to those total inequities. Given the opportunity to estimate interaction effects, it is easy to become overly focused on significance testing and lose track of intersectionality’s original critical insights and transformative aims. Conclusions of such a study should not be “there is very little intersectionality” but rather that there are meaningful inequities, they were assessed within an intersectional framework, the studied axes of marginalization were collectively important in capturing inequity patterns, and we should be addressing the needs of populations proportional to the harm they experience and challenging systems of oppression that give rise to inequities.

### 4.3 Extending the MAIHDA framework

Thus far, we have focused this tutorial on the essential elements for how to conduct and interpret results from an intersectional MAIHDA analysis of continuous or binary outcomes. There are many possible extensions to the MAIHDA approach, and some have been outlined in the growing literature. Future extensions of MAIHDA should, for instance, consider adaptations of ordinal and Poisson regression, and survival analysis. In this section, we briefly identify a few key extensions that have already been proposed.

#### 4.3.1 Eco-Intersectional Multilevel (EIM) models

Activism and research on “environmental racism” and “environmental classism” have encouraged some environmental justice scholars to take up consideration of how these social processes operate intersectionally at the community-level (Ard, 2015; Downey & Hawkins, 2008; Liévanos, 2015; Malin & Ryder, 2018; Zwickl, Ash, & Boyce, 2014). Eco-Intersectional Multilevel (EIM) analysis is an extension of the MAIHDA approach developed in the environmental justice and environmental sociology literatures to examine geospatial and social inequities in environmental hazards at the community-level (Alvarez et al., 2022; Alvarez & Evans, 2021). The core differences between MAIHDA and EIM are: (1) The level 1 units are different. While MAIHDA nests individuals at level 1, EIM nests census tracts, block groups, or other area-level descriptors such as neighborhoods at level 1. (2) The shift to different level 1 units results in some differences in theoretical models and interpretations of results. Otherwise, the approaches are similar from a modeling perspective. With growing interest in community-level inequities and critical environmental justice, the EIM approach has opened up new avenues for research and research-informed policy and activism.

#### 4.3.2 Incorporating contexts into MAIHDA

Contexts—broadly construed—have long been recognized as critical to understanding the production and determinants of health inequity (Kawachi & Berkman, 2003; Krieger, 2011). Despite this, most quantitative intersectional research has tended to examine health inequities using individual-level data without considering the separate and potentially interacting effects between those experiences and the *contexts* of the individuals in the data set (Evans, 2019c). In other words, though it is not intended based on theory, this scholarship sometimes runs the risk of de-contextualizing those same intersectional positionalities, experiences, and outcomes. This inattention to contexts is not typically shared by other types of intersectional scholarship, such as qualitative or mixed-methods intracategorical research that focuses on the lived experiences of particular social groups, grounded in specific locations and time periods (McCall, 2005; Schulz and Mullings 2006). Built into the theoretical framework is an understanding that the same social identity may be performed (or experienced, with different implications for risk exposure) differently in different contexts (Trahan, 2011).

Evans (2019c) proposed several ways to integrate consideration of contexts into intersectional MAIHDA. First, researchers can limit a study to samples from a particular social, geographic, and/or temporal context, describe the environment as part of the descriptive exercise, and then interpret quantitative results narrowly within that context. While this can produce valuable insights, it is also somewhat limited in scale, producing results that are not intended for generalizability. This approach is compatible with MAIHDA, so long as needed choices are made relating to data selection and interpretation.

Second, consideration of contexts can be incorporated into MAIHDA models by allowing for interactions (e.g., effect modification) between strata IDs and specific context variables. This could be accomplished by including key context descriptors (e.g., school and neighborhood poverty status) alongside gender, race/ethnicity, and other variables in the construction of strata IDs (Evans, 2019c). It could also be accomplished using a random slope or random coefficient term (see below).

Finally, researchers could envision a cross-classified multilevel model (CCMM) where individuals (level 1) are nested in both intersectional strata (level 2) and a context such as schools (level 2), which are cross-classified with each other. This might be done simply to test the robustness of “core” MAIHDA results when context is adjusted for (Evans, 2019a), or it might be to evaluate the relative importance of stratum and contexts (Holman, Bell, Green, & Salway, 2022; Khalaf et al., 2020; Prior and Leckie 2023). The latter, though intriguing, is more challenging to interpret. What does it mean, for instance, to say that the contributions of schools to the variance in the outcome operate semi-independently from strata positionalities, rather than being inseparable because they are a key social environment where the meaning of the strata identity is social constructed? We encourage future applications of CCMM with MAIHDA to consider and address these interpretational questions in their analyses.

#### 4.3.3 Random slopes and MAIHDA

More advanced and complex modeling structures established in multilevel methods are potentially integrable with the MAIHDA framework—including use of random slope effects where the slope coefficient on a predictor variable $x_{ij}$ is allowed to vary in its estimated magnitude across the level 2 units: $\beta_{1j}=\beta_{1}+u_{1j}$. Most simply, this additional predictor variable might not be used in the definition of the strata but could be included as a random effect. See Evans et al. (2023) for a detailed demonstration of this approach, examining how twin-vs-singleton birthweight gaps differ across intersectional strata, and for an expanded discussion of the methodological possibilities. This opens up a variety of model types to address many research questions—including in longitudinal data allowing strata to have different trajectories over time (Bell, Evans, Holman, & Leckie, 2023), and the mean outcomes of different strata to be differentially impacted by different contexts, policies, or treatments.

A further alternative is to calculate absolute risk differences (ARD) between similar strata that only differ on the exposition of interest. For instance, using MAIHDA, Zettermark et al. (2021) observed that the known association between use of hormonal contraceptives and increased risk of antidepressants use varied between intersectional strata, being more pronounced in more oppressed intersectional contexts.

## 5 Conclusion

This tutorial aimed to familiarize researchers with intersectional (and multicategorical) MAIHDA for both continuous and binary individual outcomes and to equip them to apply MAIHDA to a variety of substantive empirical questions across the social and health sciences. MAIHDA is a flexible and powerful tool for integrating an intersectional framework into quantitative study designs, and it has numerous advantages over conventional modeling approaches. We hope this method will be used in alignment with the foundational aims of intersectionality—to reveal hidden realities, expose power structures producing inequity and injustice, and critique and transform systems of oppression.

## Funding

This work was funded in part by the 10.13039/501100000269Economic and Social Research Council, grant number ES/X011313/1.

## CRediT authorship contribution statement

**Clare R. Evans:** Conceptualization, Data curation, Formal analysis, Funding acquisition, Investigation, Methodology, Project administration, Software, Supervision, Validation, Visualization, Writing – original draft, Writing – review & editing. **George Leckie:** Conceptualization, Data curation, Formal analysis, Funding acquisition, Investigation, Methodology, Software, Validation, Visualization, Writing – review & editing. **S.V. Subramanian:** Conceptualization, Methodology, Writing – review & editing. **Andrew Bell:** Software, Validation, Writing - review & editing. **Juan Merlo:** Conceptualization, Data curation, Investigation, Methodology, Writing – review & editing.

## Declaration of competing interest

The authors declare that they have no known competing financial interests or personal relationships that could have appeared to influence the work reported in this paper.

## Data Availability

The 'data' used in this paper is entirely simulated. However, for demonstration purposes it is provided as one of the Supplemental files.
